# The Role of Sanatoria as a Factor in Checking Tuberculosis

**Published:** 1907-07-13

**Authors:** 


### THE ROLE OF SANATORIA AS A FACTOR IN CHECKING TUBERCULOSIS.

Professor E. J. McWeeney, Bacteriologist to the Local Government Board for Ireland, in a paper read before the Conference of the Royal Sanitary Institute at Dublin thus sums up the part played by sanatoria in the treatment of tuberculosis:—

1. It is the only curative institution.
2. Without a place whither curable cases can be sent, the work done against tuberculosis can be at best only advisory, and therefore ineffective.
3. It provides the best centre on which popular endeavour may be focussed, and from which hopeful and hygienic streams of influence can radiate on to the community at large.
4. It may be provided out of local taxation under the existing law.

Conditions essential for the success of the sanatorium are:—

1. The reception of early cases only. When tubercle bacilli have appeared in the sputum, the case has already passed out of its earliest stage and into that of lung destruction. Modern methods, such as *x*-rays, agglutination, and diagnostic injections with Koch's old tuberculin, should be resorted to in order to assure the diagnosis before the sputum becomes bacilliferous.

2. The conduct of the institution by a genuine expert—*i.e.*, a medical man who has previously devoted all his attention for several years to sanatorium work. Much of his success will depend on his power of individualising his cases, and this cannot be done without special study.

3. The situation should be suitable and the construction specialised, with the view of (*a*) dust prevention and ready cleansing; (*b*) avoiding draughts whilst securing the maximum of fresh air; (*c*) providing arrangements for the frequent bathing of the patients, and the adoption of those hydro-therapeutic measures which are an essential portion of any serious attempt to cure the disease.

4. The patients must not be allowed to return to their unhealthy avocations and surroundings, but must be provided with suitable, light, out-of-door employment after their "cure." The admirable system devised and worked by Dr. Chapman at Clacton-on-Sea of having a market garden attached to the sanatorium, wherein the inmates can gradually acclimatise themselves to work and gain a livelihood, was referred to with warm approbation by the speaker.

While heartily concurring in these views, we are in even greater sympathy with those of subsequent speakers at the Conference who insisted on the importance, before embarking on the large expenditure involved in the provision of sanatoria, of first removing the conditions which produce the disease. As Professor Koch has rightly said: "It is the overcrowded dwellings of the poor that we have to regard as the real breeding-places of tuberculosis; it is out of them that the disease always crops up anew, and it is to the abolition of those conditions that we must first and foremost direct our attention if we wish to attack the evil at its root and wage war against it with effective weapons." What is the good of incurring large expenditure in the costly provision of sanatoria while all the time the economic factor precludes the eradication of these breeding-dens? It is because we cannot afford to prevent it that so large a proportion of our population continues to live in overcrowded one-room tenements, where a considerable proportion of them must inevitably develop consumption. Until the municipalities have removed this and the other conditions productive of the disease it will not be wise for them to dissipate their resources on costly measures which aim at dealing with the results of the evil which they must perforce permit to continue.

